# Tobacco Smoke and Inhaled Drugs Alter Expression and Activity of Multidrug Resistance-Associated Protein-1 (MRP1) in Human Distal Lung Epithelial Cells *in vitro*

**DOI:** 10.3389/fbioe.2020.01030

**Published:** 2020-09-08

**Authors:** Mohammed Ali Selo, Anne-Sophie Delmas, Lisa Springer, Viktoria Zoufal, Johannes A. Sake, Caoimhe G. Clerkin, Hanno Huwer, Nicole Schneider-Daum, Claus-Michael Lehr, Sabrina Nickel, Oliver Langer, Carsten Ehrhardt

**Affiliations:** ^1^School of Pharmacy and Pharmaceutical Sciences and Trinity Biomedical Sciences Institute, Trinity College Dublin, Dublin, Ireland; ^2^Faculty of Pharmacy, University of Kufa, Al-Najaf, Iraq; ^3^Preclinical Molecular Imaging, AIT Austrian Institute of Technology GmbH, Seibersdorf, Austria; ^4^Department of Cardiothoracic Surgery, Völklingen Heart Centre, Völklingen, Germany; ^5^Helmholtz Institute for Pharmaceutical Research Saarland, Helmholtz Centre for Infection Research, Saarbrücken, Germany; ^6^Department of Pharmacy, Saarland University, Saarbrücken, Germany; ^7^Department of Clinical Pharmacology, Medical University of Vienna, Vienna, Austria; ^8^Division of Nuclear Medicine, Department of Biomedical Imaging and Image-guided Therapy, Medical University of Vienna, Vienna, Austria

**Keywords:** COPD, ABC transporters, pulmonary drug disposition, inhalation biopharmaceutics, efflux transporters

## Abstract

Multidrug resistance-associated protein-1 (MRP1/*ABCC1*) is highly expressed in human lung tissues. Recent studies suggest that it significantly affects the pulmonary disposition of its substrates, both after pulmonary and systemic administration. To better understand the molecular mechanisms involved, we studied the expression, subcellular localization and activity of MRP1 in freshly isolated human alveolar epithelial type 2 (AT2) and type 1-like (AT1-like) cells in primary culture, and in the NCI-H441 cell line. Moreover, the effect of cigarette smoke extract (CSE) and a series of inhaled drugs on MRP1 abundance and activity was investigated *in vitro*. MRP1 expression levels were measured by q-PCR and immunoblot in AT2 and AT1-like cells from different donors and in several passages of the NCI-H441 cell line. The subcellular localization of the transporter was studied by confocal laser scanning microscopy and cell surface protein biotinylation. MRP1 activity was assessed by bidirectional transport and efflux experiments using the MRP1 substrate, 5(6)-carboxyfluorescein [CF; formed intracellularly from 5(6)-carboxyfluorescein-diacetate (CFDA)] in AT1-like and NCI-H441 cell monolayers. Furthermore, the effect of CSE as well as several bronchodilators and inhaled corticosteroids on MRP1 abundance and CF efflux was investigated. MRP1 protein abundance increased upon differentiation from AT2 to AT1-like phenotype, however, *ABCC1* gene levels remained unchanged. MRP1 abundance in NCI-H441 cells were comparable to those found in AT1-like cells. The transporter was detected primarily in basolateral membranes of both cell types which was consistent with net basolateral efflux of CF. Likewise, bidirectional transport studies showed net apical-to-basolateral transport of CF which was sensitive to the MRP1 inhibitor MK-571. Budesonide, beclomethasone dipropionate, salbutamol sulfate, and CSE decreased CF efflux in a concentration-dependent manner. Interestingly, CSE increased MRP1 abundance, whereas budesonide, beclomethasone dipropionate, salbutamol sulfate did not have such effect. CSE and inhaled drugs can reduce MRP1 activity *in vitro*, which implies the transporter being a potential drug target in the treatment of chronic obstructive pulmonary disease (COPD). Moreover, MRP1 expression level, localization and activity were comparable in human AT1-like and NCI-H441 cells. Therefore, the cell line can be a useful alternative *in vitro* model to study MRP1 in distal lung epithelium.

## Introduction

Multidrug resistance-associated protein-1 (MRP1, 190 kDa), a member of the ATP binding cassette (ABC) superfamily of transporters, is encoded by the *ABCC1* gene (Cole, 2014a). As an efflux transporter, MRP1 plays a pivotal role in physiological detoxification. Its substrates include glutathione, glucuronate, and sulfate conjugates of drugs and endogenous molecules (Cole, 2014a,b). The transporter is highly expressed in the human lung, including bronchial, bronchiolar and alveolar epithelial cells (Flens et al., 1996; Scheffer et al., 2002).

We have become interested in pulmonary MRP1 for two reasons, its impact on inhaled drugs disposition and its potential role as a target in the treatment of chronic obstructive pulmonary disease (COPD).

It has been hypothesized that MRP1 protects lung cells against toxic insults of xenobiotics and from damage induced by oxidative stress by maintaining intracellular glutathione-glutathione disulfide homeostasis (Cole and Deeley, 2006; Cole, 2014b; Nickel et al., 2016). Inhibition of MRP1 was observed to worsen cigarette smoke extract (CSE)-induced cytotoxicity *in vitro* (van der Deen et al., 2007) and pre-clinical and clinical data suggest that changes in abundance (van der Deen et al., 2006; Wu et al., 2019) or function (Budulac et al., 2010) of the transporter are associated with occurrence and severity of COPD. Moreover, recent *in vivo* data from our group showed that pulmonary distribution and clearance of the MRP1 substrate *S*-(6-(7-[^11^C] methylpurinyl)) glutathione ([^11^C]MPG), measured with positron emission tomography (PET), was significantly dependent on MRP1 abundance (Mairinger et al., 2020).

On a molecular level, the expression and activity of MRP1 in distal lung epithelium, however, remains poorly investigated. Thus, the aims of this study were to determine the expression, cellular localization and activity of MRP1 in freshly isolated human alveolar epithelial cells in primary culture and to compare these data to the human adenocarcinoma cell line NCI-H441, which present several features of human distal lung epithelium, such as an ability to form electrically tight monolayers of polarized cells. Further it has been previously proposed to be the most promising continuously growing *in vitro* surrogate of human distal lung epithelial cells (Salomon et al., 2014; Salomon et al., 2019). In addition, the influence of CSE and commonly prescribed inhaled drugs on the abundance and activity of MRP1 *in vitro* was studied.

## Materials and Methods

### Cell Culture

NCI-H441 human distal lung epithelial cells (ATCC HTB-174) were purchased from LGC Standards (Teddington, United Kingdom). Human alveolar type 2 epithelial (AT2) cells were isolated from non-tumor lung tissue obtained from patients undergoing lung surgery according to a previously published protocol (Daum et al., 2012). The freshly isolated AT2 cells were either used directly for RNA and protein isolation or left for 2 days to attach on collagen/fibronectin coated surfaces. Alternatively, cells were cultured for 8–10 days to undergo transdifferentiation into an alveolar type 1-like (AT1-like) phenotype. Primary cell culture was performed using small airways growth medium (SAGM, Lonza, Verviers, Belgium) supplemented with penicillin (100 U/ml), streptomycin (100 μg/ml), and 1% fetal bovine serum (all purchased from Sigma-Aldrich, Dublin, Ireland). Where indicated, 10 ng/ml keratinocyte growth factor (KGF, ProSpec-Tany TechnoGene, Ltd., Rehovot, Israel) was added to the culture medium to inhibit differentiation of AT2 cells into an AT1-like phenotype. The use of human tissue specimens was approved by Saarland State Medical Board (Saarbrücken, Germany). All cell types were cultured in a humidified atmosphere at 37°C in 5% CO_2_ as described in more detail by Nickel et al. (2017).

### Preparation of CSE

The smoke of two University of Kentucky research cigarettes (3R4F) was bubbled into 20 ml of RPMI 1640 medium (Biosciences, Dublin, Ireland) using a vacuum pump to generate 100% CSE. The latter was sterile filtered to remove any particulate matter and further diluted with RPMI medium to prepare 5 and 10% CSE which was used for exposure studies. Human AT1-like and NCI-H441 cells were exposed to either freshly prepared or aged CSE, which was prepared and stored at room temperature for 14 days, to investigate their effect on MRP1 abundance and activity.

### Isolation of RNA and Real-Time Polymerase Chain Reaction (q-PCR)

RNA was isolated from freshly isolated AT2 cells, which were cultured for 8–10 days to transdifferentiate into the AT1-like phenotype and NCI-H441 cells grown in six-well plates (Greiner Bio-One GmbH, Frickenhausen, Germany) using Tri-Reagent (Sigma-Aldrich) according to the manufacturer’s instructions and as described in a previously published protocol (Nickel et al., 2017). Semi-quantitative, one-step real time PCR (q-PCR) was carried out on a 7500 Real-Time PCR System (Applied Biosystems, Inc., Foster City, CA, United States) as described previously (Nickel et al., 2017) using KiCqStart predesigned primers [(Sigma-Aldrich) for *ACTB* (forward GACGACATGGAGAAAATCTG; reverse ATGATCTGGGTCATCTTCTC) and *ABCC1* (forward AGC AGAAAAATGTGTTAGGG; reverse TACCCACTGGTAATA CTTGG)].

### Immunoblot

Western blotting was carried out to investigate MRP1 abundance in AT2, AT1-like and in NCI-H441 cells. It was also used to assess the influence of different cell culture conditions [i.e., whether growing cells under air-interfaced culture (AIC) or liquid-covered culture (LCC)] on MRP1 protein level in NCI-H441 cells. In addition, the analysis was used to determine the effect of CSE, budesonide and salbutamol sulfate on MRP1 abundance in NCI-H441 cells. Cells were grown in presence of 5 or 10 μM budesonide (Mundipharma Pharmaceuticals Limited, Dublin, Ireland) or 100 μM salbutamol sulfate (Sigma-Aldrich) for up to 6 days and compared with the negative control (medium alone) or incubated with the solvent [i.e., dimethyl sulfoxide (DMSO)] when appropriate.

Confluent cell monolayers were washed twice with ice cold phosphate buffered saline (PBS) and lysed with cell extraction buffer (Life Technologies, Eugene, OR, United States) supplemented with protease inhibitor cocktail (Roche, Mannheim, Germany). Subsequently, samples were sonicated and centrifuged at 12,000 × *g* for 15 min at 4°C. The total protein amount was determined by Pierce BCA protein assay kit (Thermo Fisher Scientific, Waltham, MA, United States) according to the manufacturer’s instructions. Polyacrylamide gel electrophoresis was carried out as described previously (Nickel et al., 2017). Following transfer onto immunoblot polyvinylidine fluoride membranes, blots were blocked with washing buffer [PBS containing 0.05% Tween 20 (PBST)] supplemented with 3% (w/v) bovine serum albumin (BSA) for at least 1 h at room temperature before being incubated overnight at 4°C with rat monoclonal anti-MRP1 Ab (clone MRPr1, GTX13368, GeneTex, Inc., Irvine, CA, United States, 1:50) in PBST supplemented with 1% (w/v) BSA. The following day, membranes were washed three times and incubated with secondary anti-rat antibody (1:5000; Santa Cruz, Dallas, TX, United States) for 1 h at room temperature. Peroxidase activity was detected with Immobilon Western Chemiluminescent HRP substrate (Millipore, Carrigtwohill, Ireland). Signals were documented using a ChemiDoc system (Bio-Rad Laboratories, Hercules, CA, United States).

### Biotinylation of Cell Surface Proteins

Cell surface protein biotinylation was carried out to determine whether MRP1 is localized at the apical or basolateral membranes of polarized AT1-like and NCI-H441 cells. The analysis was not carried out in freshly isolated AT2 cells due to their unpolarized phenotype. Apical or basolateral membrane proteins of confluent, Transwell-grown cell monolayers were labeled with sulpho-NHS-biotin (Thermo Fisher Scientific), isolated and analyzed as described in a previously published protocol (Schwagerus et al., 2015).

### Immunohistochemistry and Confocal Laser Scanning Microscopy (CLSM)

NCI-H441, AT2, and AT1-like cells cultured in Lab-Tek chamber slides (Nunc A/S, Roskilde, Denmark) or on Transwell Clear inserts (either 12 mm or 6.5 mm in diameter, 0.4 μm pore size, Corning, Bedford, MA, United States) were used and processed for immunocytochemistry, as described previously (Nickel et al., 2017). Cells were fixed with 2% paraformaldehyde, incubated with 50 mM aqueous NH_4_Cl and then permeabilized with 0.1% Triton X-100. Monolayers were blocked with 2% BSA in PBS and incubated overnight with rat monoclonal anti-MRP1 Ab (1:50 dilution). The following day, cell monolayers were washed twice and incubated with Alexa Fluor 488-conjugated goat anti-rat secondary antibody (ab150165, abcam, Cambridge, United Kingdom, 1:100) for 1 h at room temperature, followed by 5 min incubation with a Hoechst 33342 solution (1 μg/ml) to counterstain nuclei. Samples were visualized on a Leica SP8 confocal laser scanning microscope (Leica Microsystems, Wetzlar, Germany) with a 63× oil immersion objective, a 488 nm diode laser and 520 nm Alexa Fluor 488 detection filter (green antibody signal) and a 405 nm diode laser in combination with a Hoechst 33342 detection filter (blue nucleic signal).

### Bidirectional 5(6)-Carboxyfluorescein Transport Studies

Multidrug resistance-associated protein-1 activity *in vitro* was determined by carrying out bidirectional transport studies of the MRP1 substrate 5(6)-carboxyfluorescein (CF) across polarized monolayers of AT1-like and NCI-H441 cells as described by Salomon et al. (2014). CF is formed intracellularly from the cleavage of its non-fluorescent diacetate conjugate (CFDA) which diffuses passively into the cells. Due to their unpolarized phenotype, transport studies could not be carried out in freshly isolated AT2 cells. Cells were grown in Transwell Clear inserts and studies were carried out only across monolayers with transepithelial electrical resistance (TEER) values exceeding 500 Ω × cm^2^. Monolayers were washed twice with pre-warmed Krebs-Ringer buffer (KRB; 116.4 mM NaCl, 5.4 mM KCl, 0.78 mM NaH_2_PO_4_, 25 mM NaHCO_3_, 5.55 mM glucose, 15 mM HEPES, 1.8 mM CaCl_2_, and 0.81 mM MgSO_4_; pH 7.4) and then pre-equilibrated with the buffer solution for 1 h in the presence or absence of the MRP1 inhibitor MK-571 (20 μM, Santa Cruz). Permeation studies were initiated by replacing the buffer solution in the donor chambers with 100 μM CFDA solution (Applied BioProbes, Rockville, MD, United States) with or without the inhibitor compound. Cells were maintained at 37°C and 200 μl samples were collected from the receptor chambers after 0, 15, 30, 45, 60, 75, and 90 min and replaced with the same volume of buffer solution. Fluorescence intensity was analyzed using an automated plate reader (FLUOstar Optima, BMG LABTECH, Offenburg, Germany) at excitation and emission wavelengths of 485 and 520 nm, respectively. TEER values were measured before and after the study to verify cell monolayer integrity. The apparent permeability coefficient (*P*_app_) was calculated using the following equation:

(1) $$P_{\text{app}}=\frac{\Delta Q/\Delta t}{A\times C_{0}}$$

where Δ*Q* is the change in the amount of CF over the designated period of time (Δ*t*), *A* is the nominal surface area of the growth supports (i.e., 1.13 and 0.33 cm^2^ in case of 3460 and 3470 Transwell inserts, respectively), and *C*_0_ is the initial concentration of CFDA in the donor fluid.

### CF Efflux Studies

Bidirectional CF efflux studies were carried out on polarized AT1-like and NCI-H441 cell monolayers grown in Transwell Clear inserts. Cell monolayers were washed twice and then incubated with KRB alone or supplemented with MK-571 (20 μM) for 1 h. Afterward, cells were loaded with 100 μM CFDA solutions (± MK-571) by adding 0.5 and 1.5 ml into the apical and basolateral chambers, respectively. Following 1 h incubation, monolayers were washed twice, and the donor solutions were replaced with the same volumes of fresh KRB alone or supplemented with the inhibitor compound. Afterward, 200 μl samples were collected from both apical and basolateral chambers every 15 min and up to 90 min and replaced with the same volumes of fresh KRB. At the end of experiments, TEER values were measured to confirm cell monolayers integrity.

Carboxyfluorescein efflux studies from AT-like and NCI-H441 cell monolayers grown in 24-well plates (Greiner Bio-One) were carried out to determine the effect of a series of inhaled drugs and CSE on MRP1 activity *in vitro*. As described above, cell monolayers were loaded with CFDA solution alone or containing either budesonide (5 or 10 μM), beclomethasone dipropionate (50 μM, Sigma-Aldrich), salbutamol sulfate (100 μM), salbutamol base (100 μM, Sigma-Aldrich), R-salbutamol HCl (100 μM, Sunovion Pharmaceuticals, Marlborough, MA, United States), S-salbutamol HCl, (100 μM, Sunovion Pharmaceuticals), terbutaline (100 μM, Sunovion Pharmaceuticals), formoterol fumarate (100 μM, Santa Cruz), L-sulforaphane (10 μM, Cayman Chemical, Ann Arbor, MI, United States), 5 or 10% CSE or the solvent [i.e., dimethyl sulfoxide (DMSO)] when appropriate. Following 1 h incubation, CFDA solutions were replaced with fresh KRB alone or supplemented with one of the above compounds and CF efflux was assessed by collecting 200 μl samples every 15 min. After 90 min, cell monolayers were washed twice with ice cold KRB and lysed in cell extraction buffer and fluorescence intensity was analyzed as described above. For standardization, the total protein concentration of whole cell lysate was determined by Pierce BCA assay (Thermo Fischer Scientific) according to the manufacturer’s instructions and the amount of CF effluxed per μg of protein was calculated.

### Data Analysis

Results are expressed as means ± SD. The significance of differences between mean values was determined either by unpaired, two-tailed Student’s *t*-test or one-way ANOVA followed by Bonferroni’s *post hoc* comparisons test. *P* ≤ 0.05 was considered significant. All experiments were carried out at least in triplicate using cells from three different passages or donors.

## Results

### Expression Analysis of MRP1/*ABCC1* in Human Alveolar Epithelial Cells

**FIGURE 1:**
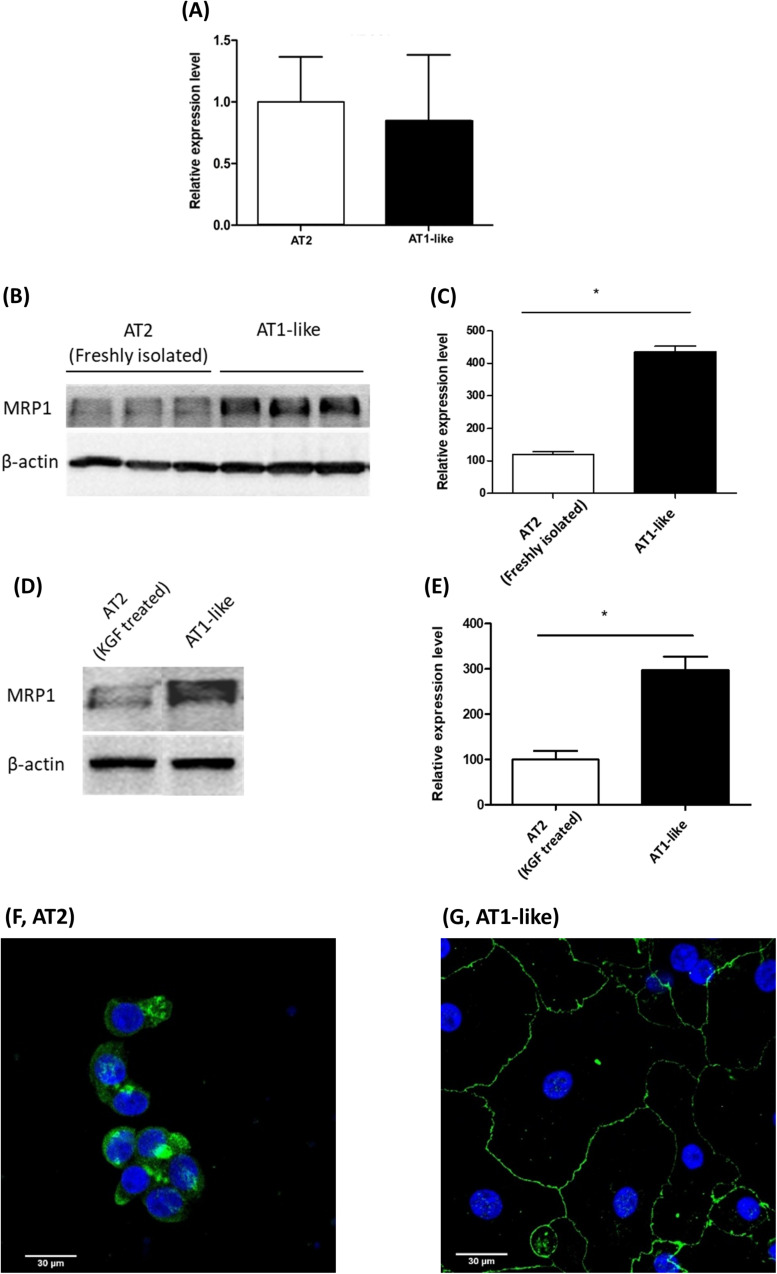
Expression of multidrug resistance-associated protein-1 (MRP1)/*ABCC1* during transdifferentiation of human primary alveolar epithelial cells. **(A)** q-PCR analysis of *ABCC1* mRNA from three donors shows stable expression upon differentiation of alveolar type 2 epithelial (AT2) cells into an alveolar type 1-like (AT1-like) phenotype. **(B,C)** Representative Western blot and corresponding densitometric analyses of MRP1 protein in alveolar cells isolated from three donors show significantly lower levels in freshly isolated AT2 cells than cells differentiated into AT1-like phenotype. **(D,E)** show lower MRP1 abundance observed in cells cultured in the presence of KGF and thus retaining their AT2 properties than in cells differentiated into AT1-like cells in the absence of the growth factor. Data are represented as means + SD, *n* ≥ 3, *P ≤ 0.01. Unpaired, two-tailed Student’s *t*-test was used. Confocal laser scanning microscopic analysis of MRP1 (green signals) in AT2 cells cultured for 2 days **(F)** was inconclusive because of the non-polarized phenotype of the cells. **(G)** In AT1-like cells, the signals detected were mainly at the basolateral cell membranes. Cell nuclei were counterstained with Hoechst 33342 (blue signals).

**FIGURE 2:**
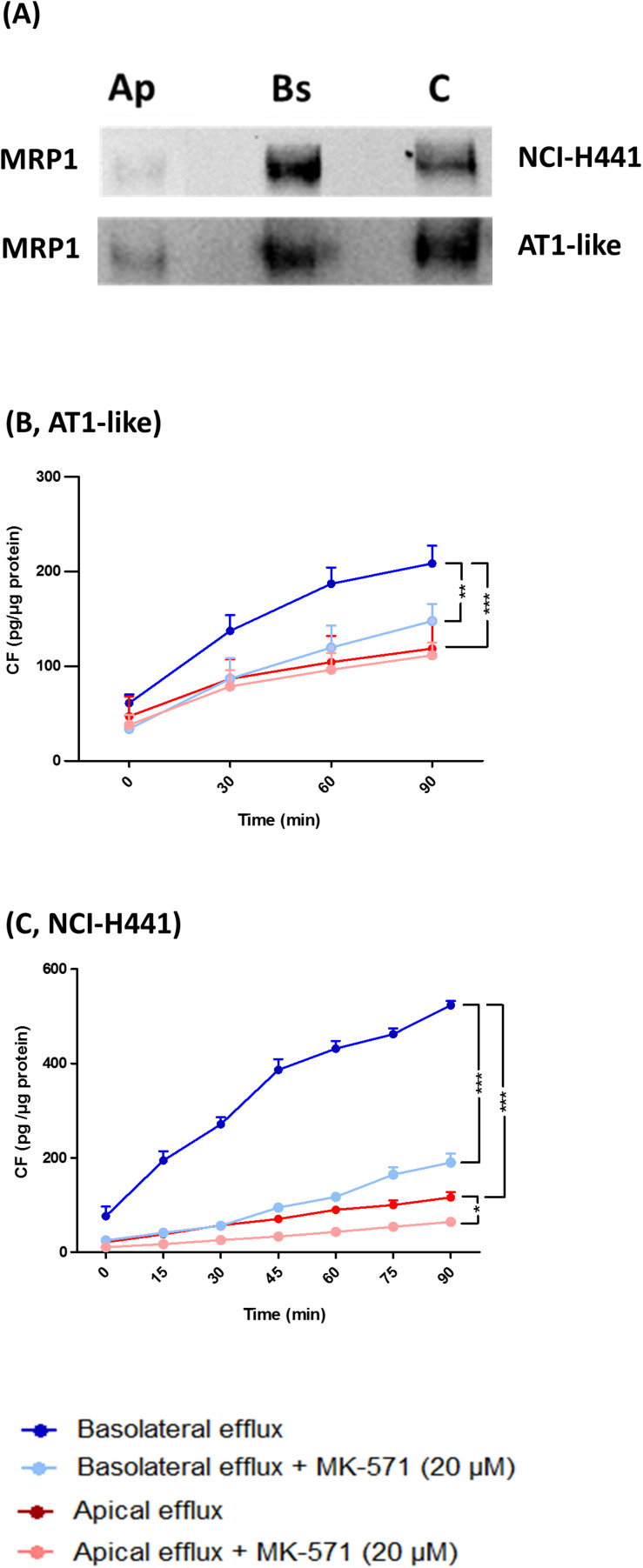
Multidrug resistance-associated protein-1 is localized to the basolateral membranes of alveolar type 1-like (AT1-like) and NCI-H441 cells. **(A)** Apical or basolateral membranes proteins of polarized, Transwell-grown NCI-H441 **(upper)** and human AT1-like **(lower)** cell monolayers were biotinylated, isolated and detected by immunoblot. Immunoblots clearly show MRP1 localization in the basolateral membranes of both cell models. Ap and Bs, indicate apically and basolaterally sulfo-NHS-biotin treated cells, respectively, and C indicates whole cell lysate. **(B,C)** show bidirectional carboxyfluorescein (CF) efflux studies from AT1-like **(B)** and NCI-H441 **(C)** cell monolayers. Consistent with the basolateral localization of the transporter, MK-571 sensitive, net basolateral efflux of CF from both cell models was observed. Data are represented as means + SD, *n* = 9, *P (0.05, **P (0.01, ***P (0.001. One-way ANOVA followed by Bonferroni’s post hocpost-hoc comparisons test was used.

Semi-quantitative real-time PCR analysis revealed *ABCC1* mRNA expression at similar levels in freshly isolated AT2 cells and cells differentiated into an AT1-like phenotype (Figure 1A). Analysis of immunoblot data, however, revealed significantly (*P* ≤ 0.01) lower MRP1 protein abundance in freshly isolated AT2 cells than in AT1-like cells from the same donors (Figures 1B,C). To exclude the probability that the lower protein levels observed in freshly isolated AT2 cells was an artifact due to cleavage of MRP1 protein by enzymes used for cell isolation, AT2 cells from the same donors were cultured in the presence or absence of 10 ng/ml KGF for at least 8 days. The growth factor has been shown to inhibit differentiation of AT2 cells into AT-like phenotype in primary culture (Demling et al., 2006). MRP1 abundance was, again, significantly (*P* ≤ 0.01) lower in cells which retained their AT2 characteristics than in cells differentiated into an AT1-like phenotype (Figures 1D,E). CLSM analysis was inconclusive in terms of MRP1 localization in unpolarized AT2 cells (Figure 1F), but MRP1 signals were primarily localized to the basolateral cell membranes of AT1-like cells grown to monolayers for at least 8 days (Figure 1G). The localization of MRP1 in the basolateral membranes of AT1-like cells was further confirmed by cell surface protein biotinylation and subsequent analysis by Western blotting (Figure 2A).

### Expression Analysis of MRP1/*ABCC1* in NCI-H441 Cells

**FIGURE 3:**
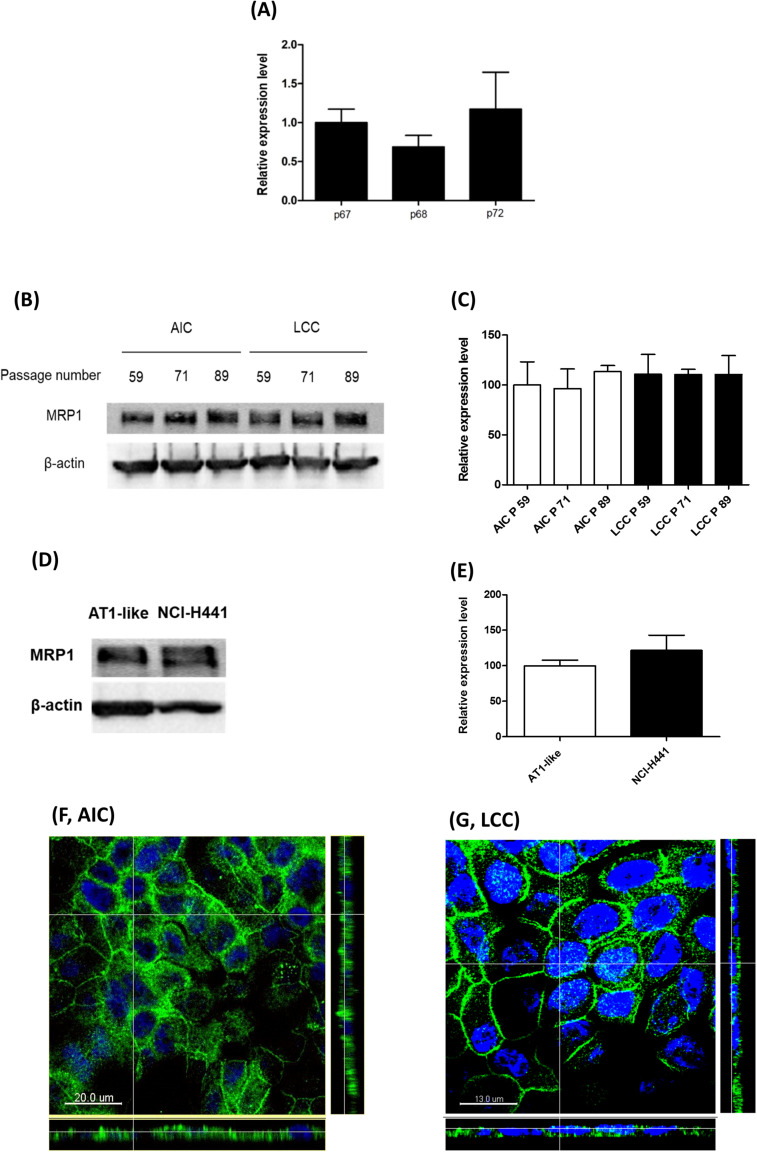
Expression of multidrug resistance-associated protein-1 (MRP1)/*ABCC1* in NCI-H441 cells. **(A)** q-PCR analysis shows stable expression of *ABCC1* mRNA across several passages, p67, p68, and p72 indicate 67th, 68^*th*^, and 72th passage numbers, respectively. **(B,C)** Western blot and densitometric analyses of MRP1 in NCI-H441 cells grown either under air-interfaced culture (AIC) or liquid-covered culture (LCC) conditions for at least 7 days show similar abundance across different passage numbers and culture conditions. **(D,E)** Western blot and densitometric analysis showing similar protein levels of MRP1 in alveolar type 1-like (AT1-like) and NCI-H441 cells. Data are represented as means + SD, *n* ≥ 3. Confocal laser scanning microscopy (CLSM) analysis of MRP1 (green signals) in NCI-H441 cells grown under AIC **(F)** and LCC **(G)** conditions show MRP1 signals primarily along the basolateral cell membranes. Cell nuclei were counterstained with Hoechst 33342 (blue signals).

Semi-quantitative real-time PCR and Western blot analyses showed stable MRP1/*ABCC1* expression across several passages of NCI-H441 cells. Neither passage number nor cell culture conditions (i.e., whether cells were grown in LCC or AIC conditions) had an influence on MRP1 abundance (Figures 3A–C). Protein levels of the transporter in the NCI-H441 cell line were comparable to AT1-like cells (Figures 3D,E). CLSM analysis of MRP1 in NCI-H441 cells grown under AIC (Figure 3F) and LCC (Figure 3G) conditions showed MRP1 signals mainly along the basolateral membranes. Cell surface protein biotinylation of Transwell-grown NCI-H441 monolayers further confirmed the basolateral localization of the protein (Figure 2A).

### MRP1 Activity in Distal Lung Epithelial Cells

**FIGURE 4:**
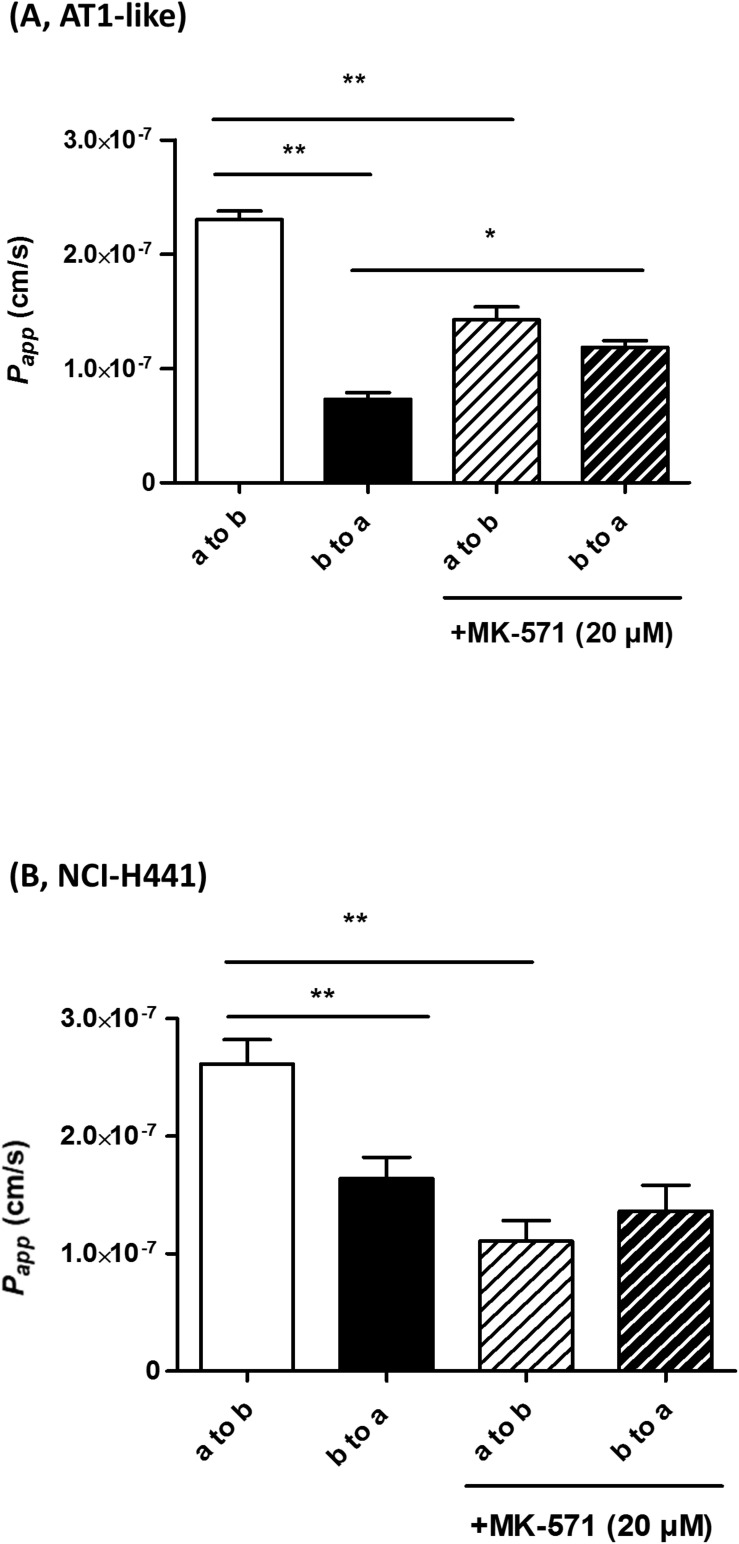
Bidirectional transport studies of carboxyfluorescein (CF) across Transwell-grown confluent monolayers of human alveolar type 1-like (AT1-like) **(A)** and NCI-H441 **(B)** cells show significant net absorption of CF, consistent with the basolateral localization of multidrug resistance-associated protein-1 (MRP1). This net apical-to-basolateral transport can be attenuated by MK-571. Data represent means + SD, *n* = 9, *P ≤ 0.05, **P ≤ 0.01. One-way ANOVA followed by Bonferroni’s *post hoc* comparisons test was used.

Multidrug resistance-associated protein-1 activity was studied *in vitro* by bidirectional transport and efflux studies of CF in polarized, Transwell-grown AT1-like and NCI-H441 monolayers, respectively. Data obtained were consistent with a basolateral localization of the transporter. A significantly (*P* = 0.001) higher amount of CF was effluxed into the basolateral receiver chamber than the apical receiver chamber from human AT1-like (Figure 2B) as well as NCI-H441 (Figure 2C) monolayers. The CF efflux was sensitive to inhibition by MK-571. Likewise, bidirectional transport studies showed MK-571-sensitive, net apical-to-basolateral transport of CF across both cell types (Figures 4A,B).

### Effect of Inhaled Drugs on MRP1 Activity and Abundance

**FIGURE 5:**
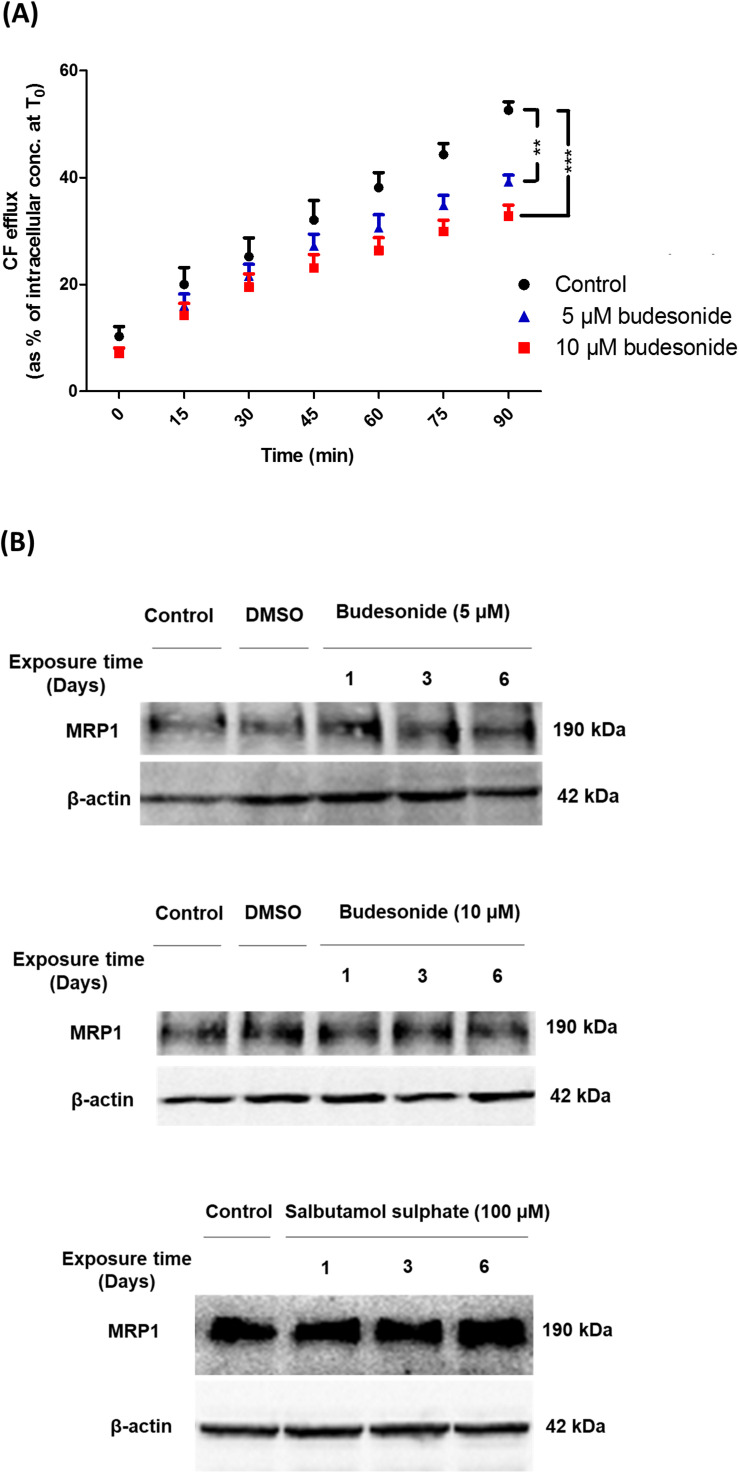
Effect of several inhaled drugs on multidrug resistance-associated protein-1 (MRP1) activity and expression. **(A)** Efflux studies from alveolar type 1-like (AT1-like) cell monolayers show significant and dose-dependent reduction of carboxyfluorescein (CF) efflux in the presence of budesonide. **(B)** Immunoblot analysis shows that treatment of NCI-H441 cells with budesonide (5 or 10 μM) or salbutamol sulfate (100 μM) for up to 6 days has no effect on MRP1 abundance. Data are represented as means + SD, *n* ≥ 6, **P ≤ 0.01, ***P ≤ 0.001. One-way ANOVA followed by Bonferroni’s *post hoc* comparisons test was used.

Carboxyfluorescein efflux studies were carried out to assess the effect of a set of commonly prescribed inhaled drugs on MRP1 activity *in vitro*. Budesonide (5 or 10 μM) decreased CF efflux from AT1-like and NCI-H441 cell monolayers in a dose-dependent manner (Figure 5A and Table 1). Similarly, beclomethasone dipropionate (50 μM) and salbutamol sulfate (100 μM) resulted in a significant reduction of CF efflux from NCI-H441 cells. Other tested bronchodilator compounds had no such effect (Table 1).

**TABLE 1:** Influence of Inhaled drugs on multidrug resistance-associated protein-1 (MRP1) activity in NCI-H441 cells.

Treatment	CF effluxed after 60 min (% of intracellular conc. at *T*_0_)
KRB	45.4 ± 11.2
KRB + DMSO	47.2 ± 7.5
Budesonide 5 μM	36.5 ± 4.3
Budesonide 10 μM	34.2 ± 7.4*
Beclomethasone dipropionate 50 μM	20.4 ± 3.04***
Salbutamol sulfate 100 μM	35.1 ± 5.9*
Salbutamol base 100 μM	41.6 ± 2.3
R-Salbutamol HCl 100 μM	41.8 ± 2.5
S-Salbutamol HCl 100 μM	37.8 ± 6.2
Formoterol fumarate 100 μM	45.3 ± 3.6
Terbutaline 100 μM	43.7 ± 1.1

Carboxyfluorescein (CF) efflux studies from NCI-H441 cells were carried out in presence of a set of beta-agonists and inhaled corticosteroids and compared with controls to assess their effect on MRP1 activity. Cells treated with transport buffer alone served as control. Data are represented as means ± SD, n ≥ 9. *P ≤ 0.05, ***P ≤ 0.001. One-way ANOVA followed by Bonferroni’s post hoc comparisons test was used.

Immunoblot analysis of NCI-H441 cells grown in the presence of either 5 or 10 μM budesonide or 100 μM salbutamol sulfate for up to 6 days showed no influence of the drugs on MRP1 abundance (Figure 5B).

### Effect of CSE and Sulforaphane on MRP1 Activity and Abundance

**FIGURE 6:**
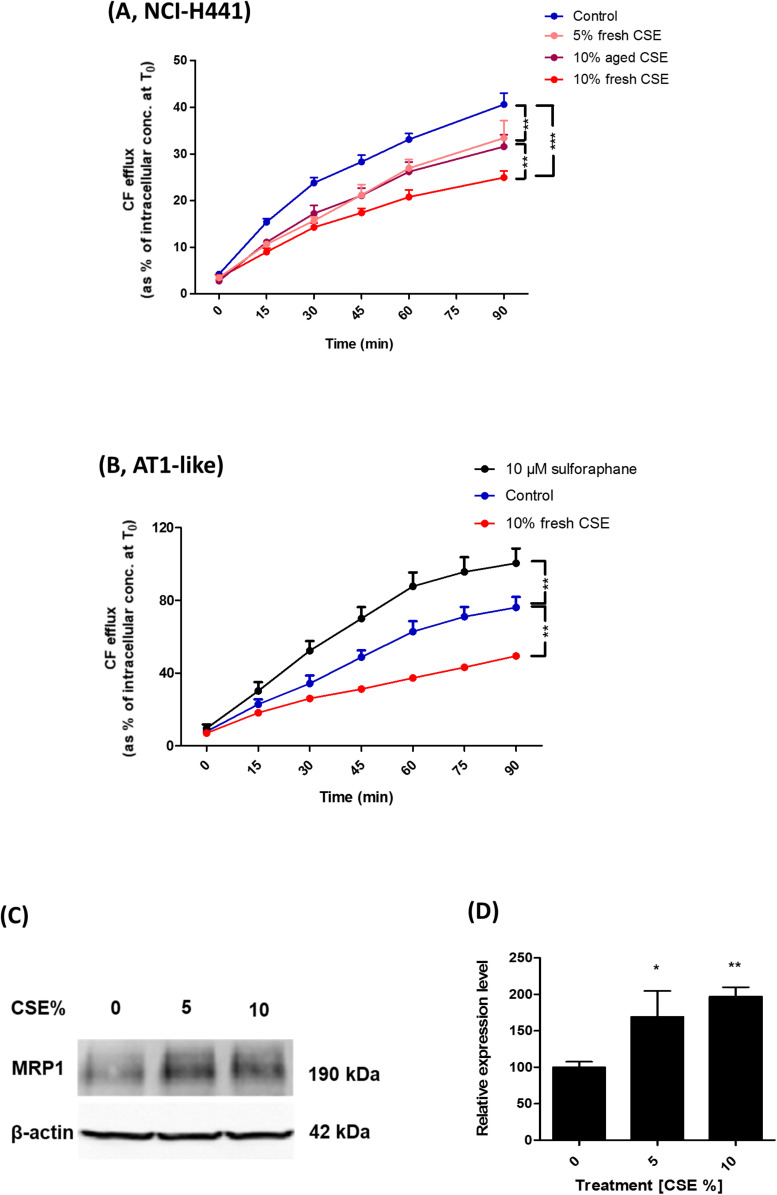
Effect of cigarette smoke extract (CSE) and sulforaphane on multidrug resistance-associated protein-1 (MRP1) activity and abundance. Exposing NCI-H441 **(A)** and alveolar type 1-like (AT1-like) **(B)** cells to CSE for 24 h results in a significant and dose-dependent reduction of MRP1-mediated carboxyfluorescein (CF) efflux from both cell types. **(C,D)** Immunoblot analysis of NCI-H441 cells exposed to CSE show a dose-dependent increase in MRP1 abundance. Data are represented as means + SD, *n* ≥ 3, *P ≤ 0.05, **P ≤ 0.01, ***P ≤ 0.001. One-way ANOVA followed by Bonferroni’s *post hoc* comparisons test was used.

Carboxyfluorescein efflux studies and immunoblot analysis were used to determine the influence of CSE on MRP1 activity and abundance in AT1-like and NCI-H441 cells. Twenty-four hours exposure to freshly prepared 10% CSE caused a significant (*P* ≤ 0.01) reduction in CF efflux from AT1-like and NCI-H441 cell monolayers (Figures 6A,B). The effect of the freshly prepared CSE was dose-dependent and more pronounced than that of aged CSE in NCI-H441 cells (Figure 6A). Interestingly, CSE exposure resulted in a significant increase in MRP1 abundance in NCI-H441 cells (Figures 6C,D).

The nuclear factor erythroid 2 related factor 2 (Nrf2)-antioxidant response elements (ARE) pathway has been previously suggested to be involved in the regulation of MRP1 expression (Ji et al., 2013). CF efflux studies were, therefore, carried out in the presence of the Nrf2 activator, sulforaphane to determine if it can improve MRP1 activity. The compound increased CF efflux from AT1-like cell monolayers (Figure 6B) but it had no influence on MRP1 activity in NCI-H441 cells (data not shown).

## Discussion

Multidrug resistance-associated protein-1 has previously been found to be the most abundant ABC transporter in human lung tissue in a study using liquid chromatography-tandem mass spectrometry for protein quantification (Sakamoto et al., 2013). The protein has also been detected in bronchial and bronchiolar epithelial cells, alveolar macrophages, seromucinous glands and nasal epithelium (Flens et al., 1996; Bréchot et al., 1998; Wioland et al., 2000). The transporter has also been detected in normal human bronchial epithelial cells in primary culture with substantial variations in the expression levels in cells obtained from different donors (Lehmann et al., 2001). However, the expression, subcellular localization, and activity of the transporter are poorly investigated in distal lung epithelial cells. Previous reports have shown differences in mRNA and protein expression levels of several membrane transporters both regarding spatial expression in different lung regions and regarding immortalized vs. primary cells (Endter et al., 2009; Salomon et al., 2012). To our knowledge, this is the first study in which the expression, subcellular localization, and activity of MRP1 is comprehensively investigated in human alveolar epithelial cells in primary culture and in the distal lung epithelial cell line NCI-H441. Data obtained revealed MRP1 to be expressed at high levels and functionally active in human distal lung epithelial cells. Immunoblots followed by densitometric analysis of samples from three donors showed a significant increase in MRP1 protein abundance upon differentiation of freshly isolated human AT2 cells into an AT1-like phenotype (Figures 1B,C). Likewise, MRP1 abundance was significantly lower in cells cultured in the presence of KGF and retaining their AT2 characteristics than in cells differentiated into AT1-like phenotype in the absence of the growth factor (Figures 1D,E). Similar results have previously been reported by Patel et al. (2008) in rat alveolar epithelial cells in primary culture. However, q-PCR analysis of *ABCC1* mRNA showed similar expression levels in AT2 cells and those differentiated into an AT1-like phenotype (Figure 1A). In fact, mRNA levels do not always strongly correlate with protein abundance due to distinct regulation controls at different stages and existence of numerous biological mechanisms that decouples gene level from protein level (Vogel and Marcotte, 2012; Fortelny et al., 2017).

Consistent with data previously reported in other lung epithelial cells (Bréchot et al., 1998; Hamilton et al., 2001), the subcellular localization experiments confirmed the transporter to be expressed primarily in the basolateral membranes of polarized AT1-like cells (Figure 1G, 2A lower panel). In NCI-H441 cells, MRP1/*ABCC1* expression was stable across several passage numbers. Moreover, the protein level and the subcellular localization of the transporter were comparable to human primary AT1-like cells (Figures 3A–G, 2A upper panel).

On a functional level, *in vitro* MRP1 activity data were also comparable between NCI-H441 cells and human AT1-like cells. Bidirectional CF efflux studies were consistent with a basolateral localization of the transporter in both cell types and the majority of substrate was effluxed into the basolateral receiver compartment. This efflux was sensitive to inhibition by MK-571 (Figures 2B,C). Small amounts of CF were also found in the apical compartment in both cell models, which may be due to paracellular transport of CF from the basolateral to the apical compartment. In addition, CF is a pan-MRP substrate and therefore a contribution of apically localized MRP2 and/or MRP4 to substrate efflux is conceivable as low *ABCC2* and *ABCC4* mRNA levels were reported previously in AT1-like cells (Endter et al., 2009). Bidirectional CF transport data were, again, comparable between human AT1-like and NCI-H441 cells. Consistent with basolateral localization of MRP1, MK-571 sensitive, net absorption of CF was observed in both cell models (Figure 4).

Multidrug resistance-associated protein-1, among other ABC transporters, plays a key role in the disposition of a wide variety of chemically unrelated drugs across various cellular and tissue barriers (Szakács et al., 2008). The transporter can, therefore, influence the pharmacokinetic profile and result in drug–drug interactions and modulation of pharmacologic activity of its drug substrates (Szakács et al., 2008; Marquez and Van Bambeke, 2011). The broad substrate spectrum together with the high abundance and functional activity of MRP1 observed at the alveolar epithelial barrier in our study suggest a potential role for the transporter in the pulmonary disposition of inhaled drug substrates. This is further supported by a recent *in vivo* study from our group in which profound differences in the pulmonary distribution and elimination kinetics of the MRP1 substrate [^11^C]MPG were observed with PET imaging between wild type and *Abcc1*^(–/–)^ rats following intratracheal aerosolisation of the radiotracer (Mairinger et al., 2020). In addition, a number of inhaled drugs have previously been reported to modulate MRP1 activity in the immortalized 16HBE14o- human bronchial cell line *in vitro* (van der Deen et al., 2008). Thus, we studied the potential interaction of a set of inhaled drugs with MRP1 in distal lung epithelial cells. Experiments carried out in human AT1-like and NCI-H441 monolayers showed a significant decrease of CF efflux in the presence of budesonide, beclomethasone dipropionate and salbutamol sulfate (Table 1 and Figure 5A). Similar findings were reported with budesonide in previous studies performed on the 16HBE14o- (van der Deen et al., 2008) and the Calu-1 (Bandi and Kompella, 2002) epithelial cell lines. To determine whether the observed effect is attributed to a reduction of MRP1 protein levels, NCI-H441 cells were grown in the presence or absence of budesonide and salbutamol sulfate for up to 6 days. Immunoblot analysis showed no change in the transporter protein level suggesting that inhaled drugs above are either inhibitors or substrates of MRP1 (i.e., competing with CF). Due to complexity of lung anatomy, determination of clinically achievable concentrations of inhaled drugs in the lung fluid and epithelial cells is extremely challenging (van der Deen et al., 2008; Mukherjee et al., 2012). Thus, the concentrations applied in our study were mainly based on previous *in vitro* studies (van der Deen et al., 2008; Mukherjee et al., 2012; Salomon et al., 2012). Reduced MRP1 activity observed with inhaled drug substrates may be a source of drug–drug interaction and variability in drug pharmacokinetics and could ultimately influence the safety and therapeutic efficacy of inhaled drugs.

Previous reports have suggested a potential role for MRP1 in smoking-related lung function loss and development of COPD. Reduced MRP1 protein levels were observed in lung tissue of COPD patients (van der Deen et al., 2006) and in an experimental rat model of COPD (Wu et al., 2019) using immunohistochemistry analysis. Moreover, the pulmonary clearance of inhaled [^99*m*^Tc] methoxyisobutyl isonitrile ([^99*m*^Tc] sestamibi), a radiolabeled MRP1 substrate, has been recorded to decrease in smokers and the decrement was attributed to modulation in MRP1 activity and expression profile (Mohan et al., 2019). The distal lung epithelium has been reported to be the initial site of development of tobacco smoke-induced diseases such as COPD (Baskoro et al., 2018). Thus, we investigated the potential impact of CSE on MRP1 activity and/or abundance in human AT1-like and NCI-H441 cells. Results showed a dose dependent reduction in CF efflux from monolayers of both cell types (Figures 6A,B). Immunoblot analysis, however, showed a dose dependent increase in MRP1 protein level upon exposure to CSE (Figures 6C,D). Given the protective role of MRP1 by extruding a variety of toxic xenobiotics out of lung epithelial cells, reduced MRP1 activity induced by CSE itself or inhaled drugs could further worsen the damage induced by tobacco smoke and may have a negative impact on the incidence and/or progression of COPD. The Nrf2-ARE pathway has been previously suggested to play a role in regulation of MRP1 expression (Ji et al., 2013). Therefore, we investigated whether the Nrf2 stimulator, sulforaphane, can improve MRP1 activity in distal lung epithelial cells. The compound had a positive impact on MRP1 activity in human AT1-like cells but not in NCI-H441 cells implying a possible beneficial effect in reversing the negative effect of CSE on MRP1 activity. The difference in response between both cell types could be, theoretically, attributed to differences in Nrf2-ARE pathway activity which requires further investigations.

## Conclusion

Multidrug resistance-associated protein-1 is functionally expressed at high levels to the basolateral membranes of human alveolar AT1-like cells in primary culture. The expression and activity profile of the transporter in NCI-H441 cells and AT1-like cells are similar and thus the cell line can be used as an *in vitro* model to study MRP1 in the distal lung epithelial barrier. Furthermore, tobacco smoke, inhaled drugs and sulforaphane can modulate MRP1 activity and/or abundance in distal lung epithelial cells *in vitro*, implying the transporter could be a novel therapeutic target of COPD.

## Data Availability Statement

The raw data supporting the conclusion of this article will be made available by the authors, without undue reservation.

## Author Contributions

CE, MS, OL, and SN: conceptualization. MS, JS, SN, and CE: data analysis. OL, CE, CC, and MS: funding acquisition. MS, A-SD, LS, VZ, JS, CC, HH, NS-D, and SN: investigation. MS and CE: project administration. HH, NS-D, and C-ML: resources. MS, SN, and CE: supervision. MS and CE: original draft. MS, A-SD, LS, VZ, JS, CC, C-ML, SN, OL, and CE: manuscript review and editing. All authors contributed to the article and approved the submitted version.

## Conflict of Interest

The authors declare that the research was conducted in the absence of any commercial or financial relationships that could be construed as a potential conflict of interest.
